# Human Neurobrucellosis with Intracerebral Granuloma Caused by a Marine Mammal *Brucella* spp.

**DOI:** 10.3201/eid0904.020576

**Published:** 2003-04

**Authors:** Annette H. Sohn, Will S. Probert, Carol A. Glaser, Nalin Gupta, Andrew W. Bollen, Jane D. Wong, Elizabeth M. Grace, William C. McDonald

**Affiliations:** * University of California, San Francisco, San Francisco, California, USA; † California Department of Health Services, Richmond, California, USA

**Keywords:** Brucella, brucellosis, cerebral granuloma, DNA sequencing, marine mammal, zoonosis, dispatch

## Abstract

We present the first report of community-acquired human infections with marine mammal–associated *Brucella* spp. and describe the identification of these strains in two patients with neurobrucellosis and intracerebral granulomas. The identification of these isolates as marine mammal strains was based on *omp2a* sequence and amplification of the region flanking *bp26*.

Brucellosis, caused by intracellular gram-negative bacteria of the genus *Brucella*, is endemic in many areas of the world. Exposure occurs through contact with infected animals, meat, or unpasteurized milk products. Neurobrucellosis is a rare, severe form of systemic infection and has a broad range of clinical syndromes (*1**–**3*). Central nervous system *Brucella* granulomas have been infrequently reported in sellar and parasellar sites and in the spinal cord (*4**–**6*).

Although a number of *Brucella* spp. cause systemic disease in humans, they have species-specific primary reservoirs. The six recognized species of *Brucella* are primarily associated with terrestrial mammals and rodents. Recently, *Brucella* has been found to cause infections in marine mammals (*7**,**8*). An expansion of the six current nomen species of *Brucella* has been proposed to include one (*B. maris*) or two (*B. pinnipediae* and *B. cetaceae*) new nomen species to categorize these strains (*7**,**8*).

To date, only one human infection with a marine mammal strain has been reported; this infection occurred in a research laboratory worker after occupational exposure (*9*). We present the first report of community-acquired human infection with marine mammal–associated *Brucella* spp. and describe the identification of these strains from two patients with neurobrucellosis and intracerebral granulomas.

## Case Reports

### Patient 1

Patient 1 was a previously healthy, 26-year-old Peruvian man who was evaluated in July 1985 for a 3-month history of periorbital pain, headaches, and periodic generalized tonic-clonic seizures. The initial neurologic examination was nonfocal, but subsequent computerized tomography scan showed a 5x5-cm enhancing mass in the left frontoparietal region associated with midline shift.

**Figure 1:**
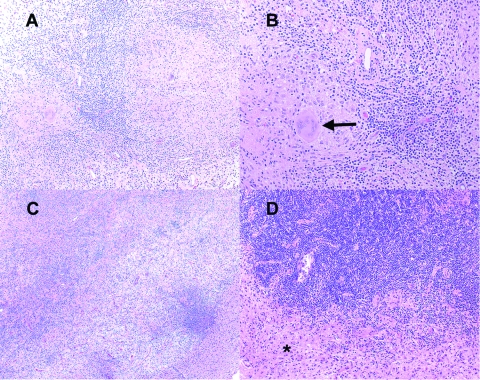
Hematoxylin and eosin–stained sections from patient 1 (panels A, B) and patient 2 (panels C, D). Note the predominantly lymphohistocytic infiltrate forming large granulomas (A, original magnification 100x); well-formed giant cells (B, arrow, original magnification 200x); lymphohistiocytic infiltrates distorting brain parenchyma and forming vague granulomas (C, original magnification 40x); and the dense astrogliosis at the interface between granulomatous inflammation and surrounding brain parenchyma (D, asterisk, original magnification 100x).

At the time of surgical biopsy, frozen section histology raised the possibility of a high-grade astrocytoma or lymphoma, prompting resection of a 3x3-cm well-circumscribed left frontal lobe mass. Final examination of pathologic specimens showed granulomas with multinucleated giant cells (Figure 1). Bacterial, fungal, and acid-fast bacilli stains were negative. Based on these pathologic findings and concern that the patient may have had tuberculosis, treatment with isoniazid, rifampin, and ethambutol was begun. Serologic tests for *Histoplasma capsulatum*, *Blastomyces dermatidis*, *Coccidioides immitis*, and *Paracoccidioides brasiliensis* were negative. *Toxoplasma gondii* serologic titers were weakly positive at 1:64.

On postoperative day 39, fungal tissue cultures became positive for a *Brucella* spp., preliminarily identified as *B. melitensis*. The patient’s antimicrobial treatment was changed to tetracycline and rifampin and was continued for 2 months. An initial serologic titer for *Brucella* was positive at 1:160 by tube agglutination assay. A follow-up serologic titer obtained in January 1986 was negative (<1:20).

Three months before his initial evaluation, the patient had immigrated to the United States from Lima, Peru. His diet included regular consumption of unpasteurized cow or goat cheese (*queso fresco*) and occasional consumption of raw shellfish (*ceviche*). He denied eating other raw or significantly undercooked meat. He had frequently swum in the Pacific Ocean from December through March but recalled no direct contact with marine mammals.

### Patient 2

**Figure 2:**
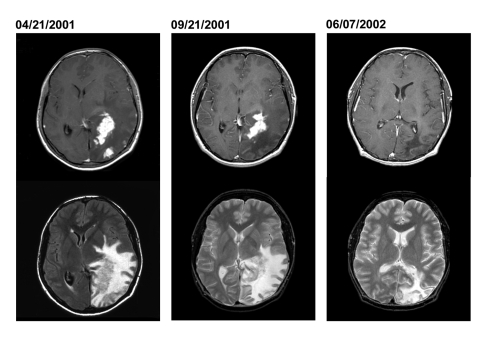
Axial MRI (magnetic resonance imaging) from patient 2 obtained when first seen in Peru (April 21, 2001), before surgical biopsy in the United States (September 21, 2001), and 7 months after start of treatment (June 7, 2002). The top images are postgadolinium-enhanced, T1-weighted images, which demonstrate resolution of one of the irregular areas of enhancement over time. The bottom images are T2-weighted images (image from April 21 is from a fluid attenuated inversion recovery [FLAIR] sequence), which demonstrate the extensive brain edema associated with these lesions. The right bottom image shows resolution of the brain edema but persistent brain atrophy.

Patient 2 was a 15-year-old Peruvian boy seen in September 2001 with a 1-year history of headaches, nausea, vomiting, and progressive deterioration in visual function. A magnetic resonance imaging (MRI) scan performed in April 2001 showed several large, irregular enhancing mass lesions involving the left occipital and parietal lobes (Figure 2). He had come to the United States in September for further evaluation. On neurologic examination, the patient had a right homonymous hemianopsia, optic nerve atrophy, and major visual impairment (left eye—20/100; right eye—20/200). Repeat MRI showed several irregular areas of enhancement in the left parietooccipital area associated with marked brain edema, left-to-right shift, and mass effect (Figure 2).

The patient was taken to surgery, where a firm avascular mass was found beneath a 0.5-cm layer of softer gliotic cortical tissue. Frozen sections showed lymphohistiocytic infiltrates with granuloma formation. Specimens for cultures and histopathologic examination were obtained. Bacterial, fungal, and acid-fast bacilli stains were negative. Final histopathologic examination showed numerous granulomas with multinucleated giant cells but no organisms (Figure 1).

Serologic test results for *Brucella*, *T. gondii*, and *Taenia soleum* were negative. On postoperative day 8, mycobacteria cultures (BacT/ALERT MP; Organon Teknika Corp., Durham, NC) showed growth of a gram-negative coccobaccillus, later confirmed as *Brucella* spp. Treatment with rifampin, doxycycline, and intravenous gentamicin was begun. After 1 week, the gentamicin was discontinued per current recommendations, and trimethoprim-sulfamethoxazole was started. Follow-up imaging 7 months later demonstrated resolution of the enhancing areas and edema, with residual areas of brain atrophy (Figure 2). The patient’s vision improved, but some visual acuity deficits persisted. Anti-brucella therapy continued for 1 year.

The patient lived in a small town 7 hours from Lima and had not traveled outside of the country before coming to the United States. His diet included regular consumption of *queso fresco* and occasional *ceviche* but no other raw meats. He reported no direct contact with marine mammals and seldom visited the Peruvian coast.

## Materials and Methods

Bacterial isolates were identified as *Brucella* spp. by using a real-time, 5′ exonuclease (TaqMan) assay, based on a well-characterized polymerase chain reaction (PCR) assay that targets a highly-conserved 223-bp region of a gene (*bcsp31*) encoding a 31-kDa immunogenic *B. abortus* protein (*10*). Amplified product was detected through the design of a dual-labeled hybridization probe, 5′-CCGGTGCCGTTATAGGCCCAATAGG (5′, 6-carboxyfluorescein label; 3′ 6- carboxytetramethylrhodamine label). Real-time detection of amplified product was performed with a LightCycler (Roche Applied Science, Indianapolis, IN) by using the following amplification parameters: 2 min at 50°C, 10 min at 95°C, and 30 cycles of 95°C for 10 s and 60°C for 30 s. Fluorescence was monitored at 530 nm.

After identifying a bacterial isolate as *Brucella* spp., we attempted further classification to the species level by using real-time PCR assays to detect *B. abortus*, *B. melitensis*, and biovar 1 of *B. suis* (*11*). We used a PCR assay targeting the *bp26* gene, performed as described by Cloeckaert et al. (*12*), to discriminate between terrestrial strains and marine mammal strains of *Brucella.*

Partial DNA sequencing of *omp2a* was also used for *Brucella* isolate classification (*13*). Amplification and sequencing of a 519-bp region of the *omp2a* gene were performed by using the primers 5′-GGGTGGCGAAGACGTTGACAA and 5′-AACCGTTGGCGCCCTATGC. Dye terminator cycle sequencing (Applied Biosystems, Foster City, CA) was performed according to the manufacturer’s recommendation; the sequencing reactions were analyzed on an ABI 377 DNA sequencer.

## Results

### Histopathology

Both cases demonstrated well-developed, granulomatous inflammation with accompanying astrogliosis. Patient 1 showed dense perivascular lymphoplasmacytic infiltrates with nonnecrotizing granulomas containing epithelioid and spindle-shaped macrophages and occasional well-formed giant cells (Figure 1). Tissue Gram, Fite, and silver methenamine stains showed no definite organisms. Patient 2 showed florid granulomatous inflammation with large areas of epithelioid and spindle-shaped histiocytes containing foci of necrosis surrounded by dense chronic inflammation (Figure 1). Giemsa, Steiner, Warthin-Starry, tissue Gram, Grocott methenamine silver, and Fite stains did not detect organisms. Giant cells were not observed in patient 2. Masson trichrome stain showed accompanying marked fibrosis.

### Molecular Microbiology

Presumptive *Brucella* isolate 01A09163 was isolated from the brain biopsy of patient 2 and was submitted to the Microbial Diseases Laboratory (the microbiology reference laboratory for the State of California) for laboratory confirmation. Identification of *Brucella* spp. was confirmed by a 5′-exonuclease assay that targets a 223-bp region of the *bcsp31* gene. Isolates confirmed as *Brucella* were then further classified as *B. abortus*, *B. melitensis*, or *B. suis* biovar 1 by the real-time PCR assays described by Redkar et al. (*11*). Strain 01A09163 tested negative by these assays, indicating that this isolate likely represented a different species of *Brucella*.

A review of Microbial Diseases Laboratory records showed a second *Brucella* strain, 85A05748, associated with an intracerebral granuloma. This strain was isolated in 1985 from the brain lesion of patient 1. Given the clinical similarities in these two cases, strain 85A05748 was tested by PCR. Like strain 01A09163, strain 85A05748 was negative in the *B. abortus*, *B. melitensis*, and *B. suis* biovar 1 assays.

To aid in the identification of these strains to the species level, a portion of the *omp2a* was amplified and sequenced. Between the two strains, the *omp2a* sequences were identical and indicated that the two strains were likely of the same species. A BLAST search of the National Center for Biotechnology Information databases established that the *omp2a* sequence of these two strains was identical to the *omp2a* sequence derived from *Brucella* sp. B2/94 (GenBank accession no. AF300819), a marine mammal strain of *Brucella* isolated from a common seal (*7*). Compared to other *Brucella* strains, one or more polymorphisms were noted in the *omp2a* sequence of 01A09163 and 85A05748. This observation suggested that strains 01A09163 and 85A05748 were most closely related to a marine mammal strain, rather than a terrestrial strain, of *Brucella*.

**Figure 3:**
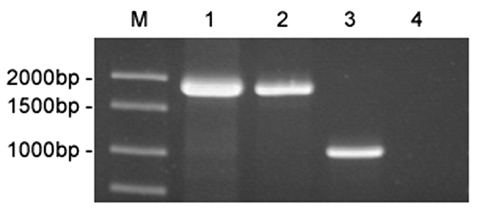
Amplification of *bp26* from marine mammal and terrestrial strains of *Brucella*. The amplification products were electrophoresed on a 1% agarose gel and stained with ethidium bromide. Lane 1, strain 01A09163; lane 2, strain 85A05748; lane 3, *B. abortus* ATCC 23448; lane 4, no template control. DNA ladder is shown in Lane M.

To verify that strains 01A09163 and 85A05748 were genetically related to strains of *Brucella* derived from marine mammals, we performed a PCR assay targeting the *bp26* gene (*12*). Marine mammal strains of *Brucella* possess an IS711 element immediately downstream of *bp26*, whereas the sequence of terrestrial strains does not. Consequently, amplification of the region surrounding *bp26* yields a much larger DNA fragment for marine mammal strains as compared to the amplification product produced by terrestrial strains of *Brucella*. As shown in Figure 3, amplification of the *bp26* gene from strains 01A09163 and 85A05748 yielded a 1,900-bp product that was diagnostic for marine mammal strains of *Brucella*. Amplification of *bp26* from a terrestrial strain, *B. abortus*, produced the expected 1,024-bp product.

## Discussion

Our report of serious central nervous system disease caused by a marine mammal *Brucella* strain confirms that these organisms can cross over from their primary hosts to humans in a community setting. *Brucella* infections have been documented in studies of marine mammals that died after being stranded ashore (*7**,**8*). Although a formal nomenclature has not yet been established, evidence is sufficient to support the expansion of the *Brucella* genus. Studies of DNA polymorphisms at the *omp2* locus indicate that more than one species may be represented in this group (*8*). When the proposed classification scheme of Cloeckaert et al. (*8*) is used, partial *omp2a* sequence suggests that the two isolates from our study are most closely related to *B. pinnipediae*, a seal strain of *Brucella*.

Despite a more than 15-year separation, these cases have a number of epidemiologic, clinical, and histopathologic similarities. Both patients had recently immigrated from Peru. They denied significant exposure to marine mammals. Neuroimaging and pathology studies were very similar. Marked granulomatous inflammation was observed in each biopsy, but histopathologic studies did not reveal the organism in tissue sections. Definitive diagnosis was subsequently made by bacterial isolation.

Notably, *Brucella* spp. antibody was not elevated in patient 2. Despite his prolonged duration of illness and extensive central nervous system involvement, the patient’s steroid medications, unknown host factors, or low immunogenicity of marine mammal strains may be responsible for his lack of serologic response, emphasizing the importance of direct isolation.

Neurobrucellosis develops in <5% of patients with *Brucella* infection (*1*). The most frequent clinical syndromes associated with acute infection are meningitis or meningoencephalitis (*1**–**3*). Mass lesions within the brain parenchyma are extremely uncommon but have been documented radiographically (*6*) and pathologically (*14*). Patient 1 underwent granuloma resection before medical therapy and did not have relapse of infection. Patient 2 was treated with medical therapy. Such infections, despite widespread central nervous system extension, could possibly be treated medically, reducing potential long-term neurologic complications of resection. Furthermore, the indolent nature of these infections suggests that early detection and treatment could prevent the long-term consequences of a chronic intracranial inflammatory process.

These cases raise important issues about the epidemiology of human *Brucella* infection related to transmission of nonterrestrial strains. In the absence of a direct association with marine mammals, more traditional zoonotic sources of infection may be involved. Experimental infection of dairy cattle with a marine mammal strain of *Brucella* has been documented (*15*). Given the extensive Peruvian coastline, marine mammal strains of *Brucella* could conceivably have been transmitted to domestic animals and wildlife that reside nearby.

What degree of pathogenicity these strains may have in human infection is unclear. Further study of infection in both humans and marine mammals is needed to characterize the spectrum of disease and the potential for communicability. Such studies may be facilitated by the implementation of new laboratory tests to rapidly identify marine mammal strains of *Brucella*.
